# circKIF4A promotes tumorogenesis of glioma by targeting miR-139-3p to activate Wnt5a signaling

**DOI:** 10.1186/s10020-020-00159-1

**Published:** 2020-04-08

**Authors:** Long-Wei Huo, Ya-Fei Wang, Xiao-Bin Bai, Hu-Lin Zheng, Mao-De Wang

**Affiliations:** 1 Department of Neurosurgery, The First Affiliated Hospital of Xi’an Jiao Tong University, No. 277 Yanta Xi Road, Xi’an, 710061 Shaanxi Province People’s Republic of China; 2 Department of Neurosurgery, Yulin First Hospital Affiliated to Xi’an Jiao Tong University, Yulin, 719000 People’s Republic of China

**Keywords:** circKIF4A, Glioma, miR-139-3p, Wnt5a, Proliferation, Migration

## Abstract

**Background:**

Glioma has the characteristics of high incidence and mortality, and is a common malignant tumor of the central nervous system. Circular RNAs (circRNAs) have been reported to play vital roles in progression of cancer including glioma, and circKIF4A is up-regulated in glioma tissues. However, its role and mechanisms in gliomas are unclear.

**Methods:**

circKIF4A and miR-139-3p were determined by qRT-PCR. Transwell assay, wound-healing assay, cell colony formation and flow cytometry were performed to measure cell invasion, migration, proliferation and apoptosis. Western blotting was used to evaluate Wnt/β-catenin pathway-related protein. Luciferase reporter assays confirmed the relationship among circKIF4A, miR-139-3p and Wnt5a. Sphere formation was performed to measure the ability of glioma-initiating cells (GICs) spheroid formation. A nude mouse xenograft model was established and immunohistochemical staining was used to detect Ki-67 and Wnt5a levels.

**Results:**

circKIF4A and Wnt5a were up-regulated and miR-139-3p was down-regulated in both glioma cells and tissues. circKIF4A promoted Wnt5a expression by sponging miR-139-3p. Knockdown of circKIF4A inhibited the colony formation ability, migration and invasion, and promoted the apoptosis of glioma cells by regulating miR-139-3p. Knockdown of circKIF4A inhibited Wnt/β-catenin signaling pathway and proliferation-related signal via miR-139-3p. Furthermore, knockdown of circKIF4A or overexpression of miR-139 suppressed the ability of sphere formation of GICs and inhibitd Wnt/β-catenin signaling pathway and proliferation-related signal in GICs. Additionally, depletion of circKIF4A decreased the expression level of Wnt5a and Ki-67, inhibited tumorigenesis in xenograft modes.

**Conclusion:**

circKIF4A was overexpressed in glioma, and knockdown of circKIF4A suppressed glioma progression via miR-139-3p/Wnt5a axis. The results indicated that circKIF4A may be a potential target for clinical treatment of glioma.

## Background

Gliomas are primary malignant brain tumors due to carcinogenesis of brain and spinal glial cells (Daniel et al. 2019). Pathologically, gliomas are classified as grade I-IV according to World Health Organization (WHO) criteria, with grades I-II considered to be low-grade gliomas (LGG) and grades III-IV high-grade glioma (HGG). WHO class IV glioma, also known as glioblastoma or glioblastoma multiforme (GBM). GBM was found to be the most common malignant primary brain tumor, accounting for 16% of all primary brain tumors and 54% of all gliomas (Chuntova et al. 2018), with high morbidity, high recurrence rate, high mortality and low cure rate (Chen et al. 2018). Although surgical techniques and adjuvant therapy have evolved over the decades, the treatment and prognosis of gliomas still face significant challenges (Tomiyama and Ichimura 2019). This undesirable result is due in part to the cell-autonomous functions of therapeutically resistant glioma-initiating cells (GICs) (Figueroa et al. 2017). Therefore, an in-depth study of the molecular mechanisms associated with glioma is of great value in the development of potential glioma targeted therapies.

Wnt/β-catenin signaling pathway is critical in cell proliferation, migration, invasion and angiogenesis, and is closely related to various tumorigenesis. In canonical Wnt/β-catenin pathway, when Wnt signal is activated, Wnt protein binds to Frizzleds (FZD), under the action of Disheveled (Dvl), it phosphorylates and accumulates a large amount of β-catenin, and then binds to T cell factor/lymphoid enhancer-binding factor (TCF/LEF) transcription complex to mediate the activation of a series of Wnt target genes such as cyclin D1 and c-Myc, thereby regulating cell differentiation and proliferation (Chu et al. 2019; de Sousa and Vermeulen 2016). Abnormal expression of the Wnt/β-catenin signaling was reported to cause the cell proliferation and inhibit the cell apoptosis in glioma, thereby promoting the progression of glioma (He et al. 2019). Wang et al. found that the Wnt/β-catenin signaling pathway is related to stemness, self-renewal, and radiation resistance of GICs (Wang et al. 2017). Wnt5a is one of the important members of the Wnt family, and more and more studies have shown that changes in Wnt5a expression are closely related to the progression of glioma. Masayuki Kamino et al. found that Wnt5a was increased in gliomas and its expression was involved in invasive activity and transcription of MMP-2 (Kamino et al. 2011). Binda E et al. confirmed that Wnt5a could improve the invasion of glioma cells, and knocking down Wnt5a inhibited the intracranial invasion of glioma and increase the survival rate of mice (Binda et al. 2017). Obviously, it is of great significance to explore the molecular mechanisms of Wnt5a-mediated β-catenin signaling pathways regulating glioma.

Circular non-coding RNA (circRNA) is a special non-coding RNA molecule with covalent bond forming ring structure discovered in recent years. It was discovered by Sanger et al. (Sanger et al. 1976) in the study of viroid in 1976 and put forward the concept for the first time. With the rapid development of molecular biology and molecular sequencing technology, the potential characteristics and functions of circRNA have been gradually discovered. Some studies (Rybak-Wolf et al. 2015) indicated that circRNA is abundant in the nervous system, showing the characteristics of dynamic expression, and may participating the proliferation, metastasis and other biological processes of glioma (Liu et al. 2019a), which is expected to become a new diagnostic marker of glioma, and providing new ideas and directions for treatment. For example, it has been found that the higher circ_0079593 level in glioma tissues is associated with higher WHO grades, larger-tumor volumes and poor survival in glioma patients, suggesting that circ_0079593 may be considered an independent prognostic predictor of glioma patients (Qu et al. 2019). circPCMTD1 gene is highly expressed in gliomas, promoting the proliferation, migration and invasion of glioma cell line, and circPCMTD1 maybe a potential target for glioma treatment (Zheng et al. 2019). Renjie Wang et al. (Wang et al. 2018a) used microarray technology to detect the differential expression of circRNAs in GBM, and found that the expression level of circKIF4A (hsa_circ_0090956) in glioma tissue was increased. However, its specific molecular mechanism is still unclear.

circRNA acts as a sponge of microRNA, which leads to the loss of miRNA function, accompanied by an increased gene target (Wang et al. 2016; Li et al. 2019). microRNAs (miRNAs) are an important class of non-coding small RNAs that regulate target gene expression at the post-transcriptional level and play a vital role in many pathological processes. Abnormal expression of miRNAs is closely related to the development of glioma, which is a potential therapeutic target for glioma (Zhou et al. 2018). miR-139-3p has been reported to be low expression in multiple cancers such as colorectal cancer, head and neck cancer, and hepatocellular carcinoma, which may act as a tumor suppressor (Kanaan et al. 2013; Wang et al. 2019). Yonemori M et al. (Yonemori et al. 2016) found that miR-139-3p targeting MMP11 to inhibit the invasion and migration of bladder cancer cells. Sannigrahi MK1 et al. (Sannigrahi et al. 2017) confirmed that miR-139-3p can inhibit HPV-16 proteins and revive key tumor suppressor proteins p53, p16, and p21, thereby inhibiting proliferation and migration in HPV-16 positive cells. Studies have shown that miR-139-3p expression level was reduced in glioma specimens, and overexpression of miR-139-3p inhibited the migration, invasion and proliferation of glioma cells (Tian et al. 2019). However, little research has been done on the molecular mechanism by which miR-139-3p regulates the development of glioma, which requires further research. We found through bioinformatics analysis that the circKIF4A and Wnt5a sequences have potential binding sites to the miR-139-3p sequence. Therefore, the interaction of circKIF4A, miR-139-3p and Wnt5a in glioma is worthy of our comprehensive investigation.

In this study, we found that circKIF4A and Wnt5a were over-expressed, and miR-139-3p was down-expressed in glioma cell lines and tissues. circKIF4A knockdown inhibited glioma cells proliferation, migration and invasion, circKIF4A knockdown suppressed tumor growth likewise in nude mice. Additionally, circKIF4A acted as a miR-139-3p sponge to enhance its target gene Wnt5a, which activated Wnt/β-catenin signaling pathway and cascades of proliferation-related signal. circKIF4A also promoted the maintenance of GICs by regulating miR-139-3p/Wnt5a signaling pathway. circKIF4A may serve as a potential target for glioma treatment.

## Methods

### Human tissue samples

32 pairs of adjacent non-tumor tissues and glioma samples were collected from the First Affiliated Hospital of Xi’an Jiao Tong University. All tissues were received at surgery and directly preserved in liquid nitrogen. The survey and experiments have obtained patients’ consent and been approved by the Ethic Committee for Clinical Research of the First Affiliated Hospital of Xi’an Jiao Tong University.

### Cell culture

Normal human astrocytes (NHAs) were obtained from Lonza (Basel, Switzerland). NHAs cells were cultured in astrocyte growth medium with 5% FBS, ascorbic acid, rhEGF, insulin, L-glutamine and GA-1000. Human glioma cell lines LN229, A172, SHG44 and U251 were obtained from the Chinese Academy of Sciences Cell Bank (Shanghai, China). The four cell lines were cultured in high-glucose Dulbecco’s Modified Eagle medium (DMEM; Invitrogen, Carlsbad, CA, USA) with 10% fetal bovine serum (FBS; Gibco, Bethesda, MD, USA) and antibiotics (100 units/mL penicillin and 100 mg/mL streptomycin). All the cells were incubated in 5% CO_2_ at 37 °C.

### Cell transfection

miR-139-3p mimics, miR-139-3p inhibitor and negative control (NC), sh-NC, sh-circKIF4A-1, sh-circKIF4A-2 and sh-circKIF4A-3 were all obtained from Genepharma (Shanghai, China). Lipofectamine 2000 (Invitrogen, Carlsbad, CA, USA) was used for transfection of cells according to the manufacturer’s instructions. The cells were cultured for 48 h after transfection and harvested for the following experiments.

### Quantitative real time-polymerase chain reaction (qRT-PCR)

The RNAs were extracted from cells or tissues by TRIzol reagen (Invitrogen, CA, USA), and reversely transcribed into cDNAs using PrimeScript RT kit (TaKaRa, Japan). According to protocol of the manufacturer’s instructions, qRT-PCR was performed using the SYBR Green PCR Master Mix kit (TaKaRa, Japan) and the ABI7500 System (Applied Biosystems, CA, USA). PCR amplification was conducted as follows: initial denaturation at 95 °C for 30s, 40 cycles of 95 °C for 22 s 60 °C for 30s. U6 and GAPDH were used as controls. The following primer sequences were used: circKIF4A forward 5′-TCCCCAGGCTACACTAATGG-3′, reverse 5′-ACTGTGGGCACCATTCTAGG-3′; miR-139-3p forward 5′-TGGAGACGCGGCCCTGT-3′, reverse 5′-GTCGTATCCAGTGCAGGGTCCGAGGTATTCGCACTGGATACGACACTCCA-3′; Wnt5a forward 5′-CCAACTGGCAGGACTTTCTC-3′, reverse 5′-CCTGCCAAAAACAGAGGTGT-3′; U6 forward 5′-CTCGCTTCGGCAGCACA-3′, and reverse 5′-AACGCTTCACGAATTTGCGT-3′ and GAPDH forward, 5′-CCAGGTGGTCTCCTCTGA-3′ and reverse 5′-GCTGTAGCCAAATCGTTGT-3′. Relative expression levels were calculated by the 2^−ΔΔCT^ method.

### Western blot assay

The proteins from cells or tissues were exacted with RIPA lysis buffer (Sigma-Aldrich, St Louis, MO). The concentration of proteins was measured by BCA assay kit (Bio-Rad Laboratories, Hercules, CA, USA). The 10% SDS-PAGE isolated protein was transferred to the PVDF membrane (Millipore, Bedford, MA, USA). Blocked the membranes at room temperature with 5% non-fat milk for 2 h. Then, the membranes were incubated in specific primary antibodies including Wnt5a (1:1000, ab229200, Abcam, USA), GSK-3β (ab93926,1:500, Abcam, USA), β-catenin (1:2000, ab6302, Abcam, USA), p-β-catenin (1:500, ab27798, Abcam, USA), c-Myc (1:1000, ab32072, Abcam, USA), cyclinD1(1:1000, ab40754, Abcam, USA), Bcl2 (1:800, ab32124, Abcam, USA), Bax (1:1000, ab32503, Abcam, USA), and GAPDH (1:5000; ab181602, Abcam, USA) at 4 °C overnight. The membranes were washed with 0.1% PBST three times for 5 min each, incubated in HRP-labeled secondary antibodies for 1 h, and washed three times. Chemiluminescent ECL Plus reagents (Pierce, USA) were added to visualize the reaction products.

### Dual luciferase reported assay

The circKIF4A or Wnt5a 3’UTR with miR-139-3p binding sites (wild type) sequences were inserted into the pGL3 plasmid vector (Promega, Wisconsin, USA), forming circKIF4A-WT and Wnt5a-MUT luciferase reporter plasmids. At the same time, the mutant sequence (MUT) of circKIF4A or Wnt5a 3’UTR was introduced to construct the structures of circKIF4A-MUT and Wnt5a-MUT, respectively. LN229 and A172 cells were first cultured in 24-well plates, and then co-transfected with pGL3 plasmid vectors carrying mutated or wild circKIF4A or Wnt5a 3’UTR sequences together with miR-139-3p inhibitor or miR-139-3p mimics. 48 h after transfection, the double luciferase reporter assay system (Promega, Madison, WI, USA) was used to measure luciferase content.

### Colony formation assay

The cells were placed in 6-well plates and incubated for 14 days at 37 °C. 4% paraformaldehyde was used to fix colony cells for 15 min, followed by 0.1% crystal violet to stain at room temperature. The number of colony cells were imaged and counted under an optical microscope (Nikon, Japan).

### Wound healing assay

Cell migration was observed using a wound healing assay. Briefly, when the transfected cells were maintained in a 6-well plate, achieving 90–95% confluence, scratches were generated using the micropipette tips. The wound state was observed at 0 h and 24 h after scratching with an X71 inverted microscope (Olympus, Tokyo, Japan).

### Transwell assay

Cell migration and invasion were performed with transwell chambers (BD Biosciences, USA). The cells were seeded in the upper layer of a transwell chamber. Cell culture medium supplemented with 20% FBS was filled in the lower chamber. After cultured 48 h, the upper chamber cells were cleared, and the lower layer of migrated cells were stained with 0.5% crystal violet (Sigma-Aldrich; Merck KGaA). The invasion assay was performed in the same way as the migration assay, however, the cells were seeded in the upper chamber of medium coated with Matrigel. The migrated and invaded cell numbers were counted under a light microscope (Nikon, Japan).

### Flow cytometry analysis

For cell apoptosis assays, transfected cells were harvested and resuspended in PBS. The cells concentration was adjusted to 1 × 10^6^ cells/mL, and then double stained with 5 μL Annexin V-FITC and 5 μL propidium iodide (PI) for 15 min. Then, the apoptosis cells were analyzed by flow cytometry (BD Biosciences, San Diego, CA, USA).

### Isolation of GICs

GICs were isolated from previously described glioma surgical specimens according to reported experimental methods (Figueroa et al. 2017). Briefly, glioma specimens were dissociated and cultured in serum-free NSC medium, consisting of Dulbecco’s Modification of Eagle’s Medium/Ham’s F12 with 2% B-27 (Invitrogen, Carlsbad, CA, USA), 20 ng/mL bFGF (Sigma-Aldrich, St. Louis, Missouri, USA), 20 ng/mL EGF (Sigma-Aldrich, St. Louis, Missouri, USA).

### Sphere formation assay

For sphere formation assays, cells were seeded in 6-well ultralow attachment plates at a density of 1 × 10^3^ cells/mL. Add Dulbecco’s modified Eagle’s medium containing 2% B27 supplement (Invitrogen, Carlsbad, CA, USA), 1% N2 supplement (Invitrogen, Carlsbad, CA, USA), 100 ng/mL epidermal growth factor (Invitrogen, Carlsbad, CA, USA), 20 ng/mL human platelet growth factor (Sigma-Aldrich, St. Louis, Missouri, USA), and 1% antibiotic-antimycotic (Invitrogen, Carlsbad, CA, USA). Then incubate in 37 °C, 5% CO_2_ and humidified atmosphere of 95% air. After 7 days, single spheres were chosen and counted.

### Nude mouse xenografts

Male BALB/C-nu/nu mice (6-week-old) were purchased from the SJA Laboratory Animal Co., Ltd. (Hunan, China), and were bred under SPF conditions. Subcutaneous injection of A172 and LN229 cells that stably expressing sh-circKIF4A into BALB/c nude mice. Tumor volume was measured every 5 days, and the tumor volumes were counted using the formula V  =  0.5* larger diameter *(smaller diameter) ^2^. Thirty days later, the tumors were removed and weighed from the sacrificed mice. In this study, the protocol was approved by the Committee on the Ethics of Animal Experiments of the First Affiliated Hospital of Xi’an Jiao Tong University.

### Immunohistochemical (IHC) staining

For immunohistochemical analysis of Ki-67 and Wnt5a proteins, paraffin-embedded glioma patients or mice tumor tissues were cut into 5-μm-thick slides. After being baked for 2 h at 60 °C, the tissues were immersed in xylene for deparaffinization and decreasing concentrations of ethanol for rehydration. Then, the slides were incubated with the Wnt5a primary antibodies (1:200, ab229200, Abcam, USA) and Ki-67 primary antibodies (1:200, ab15580, Abcam, USA) for 12 h at 4 °C. Next day, the slides were washed with PBS and incubated with biotinylated secondary antibody for 2 h at room temperature. Finally, according to the manufacturer’s instructions, slides were treated with Immunopure Metal enhanced DAB substrate kit (Pierce, Rockford IL).

### Statistical analysis

Statistical analyses using SPSS 19.0 software (SPSS Inc., USA). Student’s *t*-test was used to examine the differences between two groups. And one-way analysis of variance (ANOVA) was used to examine the differences multiple groups. Spearman correlation analysis was performed to analyze the correlation among circKIF4A, miR-139-3p, and Wnt5a in glioma samples. All data were presented as the mean ± SD. *P*-value < 0.05 was considered statistically significant.

## Results

### circKIF4A and Wnt5a are up-regulated and miR-139-3p is down-regulated in glioma cell lines and tissues

**Fig. 1:**
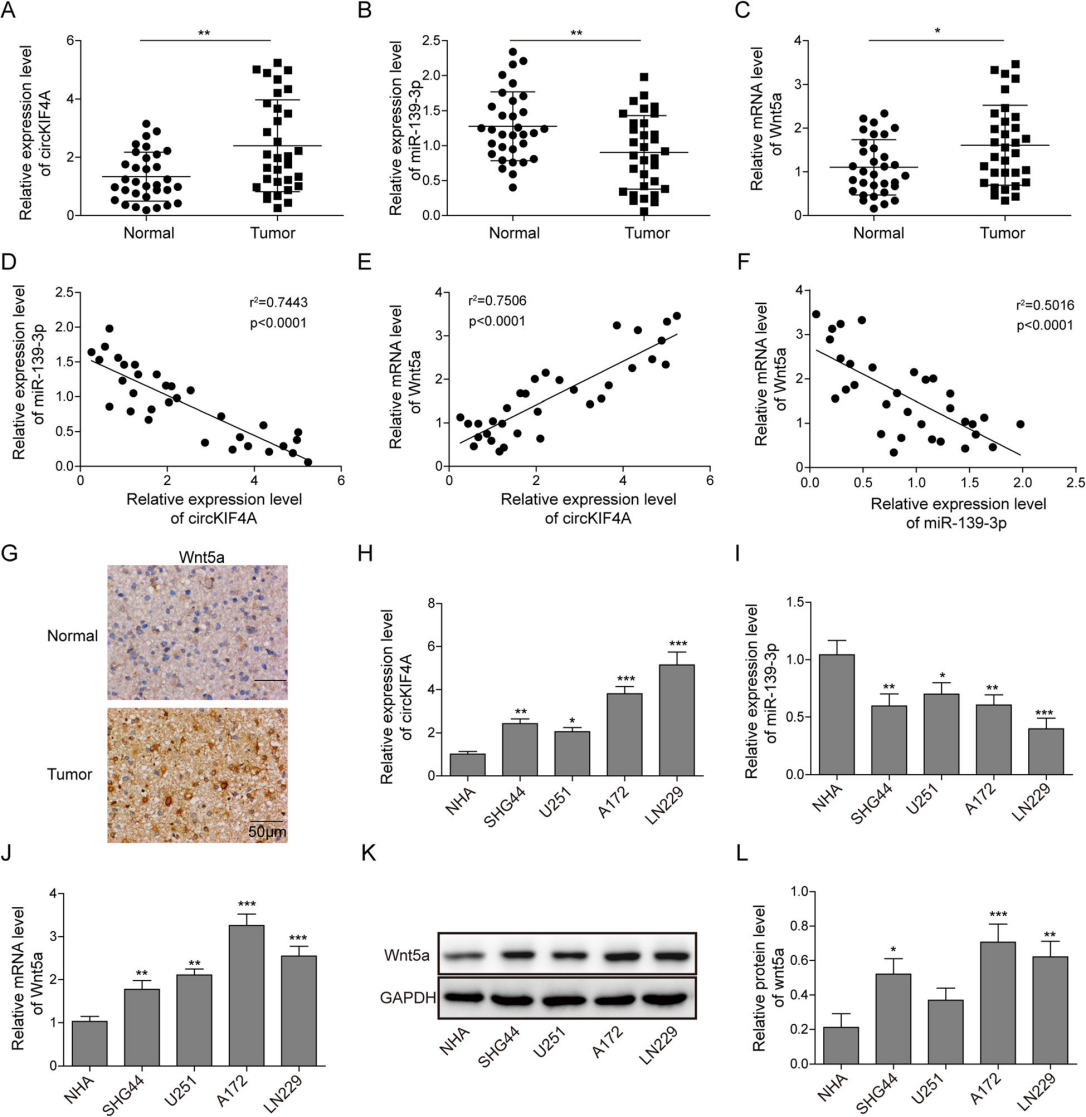
Expression of circKIF4A, miR-139-3p and Wnt5a in several glioma cell lines and tissues. The expression level of circKIF4A (**a**) miR-139-3p (**b**) and Wnt5a (**c**) was detected by qRT-PCR in glioma tissues and adjacent normal tissues. The correlation between circKIF4A and miR-139-3p (**d**), circKIF4A and Wnt5a (**e**), miR-139-3p and Wnt5a (**f**) expression were determined by Spearman correlation analysis. **g** The protein level of Wnt5a was detected by immunohistochemical in glioma tissues and adjacent normal tissues. The expression level of circKIF4A (**h**) miR-139-3p (**i**) and Wnt5a (**j**) was detected by qRT-PCR in four glioma cell lines including A172, SHG44, U251 and LN229, as compared with NHA cell line. **k** The protein level of Wnt5a was detected by Western blotting in glioma cell lines and NHA cell line. **l** The quantification of Wnt5a relative protein level as a ratio to that of GAPDH (internal control). *p < 0.05, **p < 0.01, ***p < 0.001

To investigate the roles of circKIF4A, miR-139-3p and Wnt5a in glioma, we first detected the expression of circKIF4A, miR-139-3p and Wnt5a in 32 pairs of glioma and adjacent normal tissues. Compared with normal ones, a higher level of circKIF4A and Wnt5a were detected, and a lower level of miR-139-3p was detected in glioma tissues (Fig. 1a-c). Spearman correlation analysis was performed to calculate the correlation among circKIF4A, miR-139-3p, and Wnt5a levels in glioma samples. circKIF4A was inversely correlated with miR-139-3p expression and positively correlated with Wnt5a expression (Fig. 1d, e). Meanwhile, miR-139-3p was negatively correlated with Wnt5a expression (Fig. 1f). Additionally, immunohistochemical experiments showed that the expression of Wnt5a was increased significantly and located mainly in the cytoplasm or extracellular in glioma tissues (Fig. 1g). Then, qRT-PCR was used to monitor the expressions of circKIF4A, miR-139-3p and Wnt5a in NHA and four glioma cell lines (A172, SHG44, U251 and LN229). The results demonstrated that the expressions of circKIF4A and Wnt5a were increased, and miR-139-3p level was decreased in glioma cell lines compared to NHA control cell (Fig. 1h-j). Moreover, the protein expression of Wnt5a was also increased in glioma cell lines, compared with that in NHA cells (Fig. 1k, l). These results suggested that abnormal expression of circKIF4A, miR-139-3p and Wnt5a may be involved in glioma progression. Among the four glioma cell lines, circKIF4A was expressed at higher levels in the A172 and LN229 cell lines, so the A172 and LN229 cell lines were used for further study.

### circKIF4A promotes Wnt5a expression by sponging miR-139-3p

**Fig. 2:**
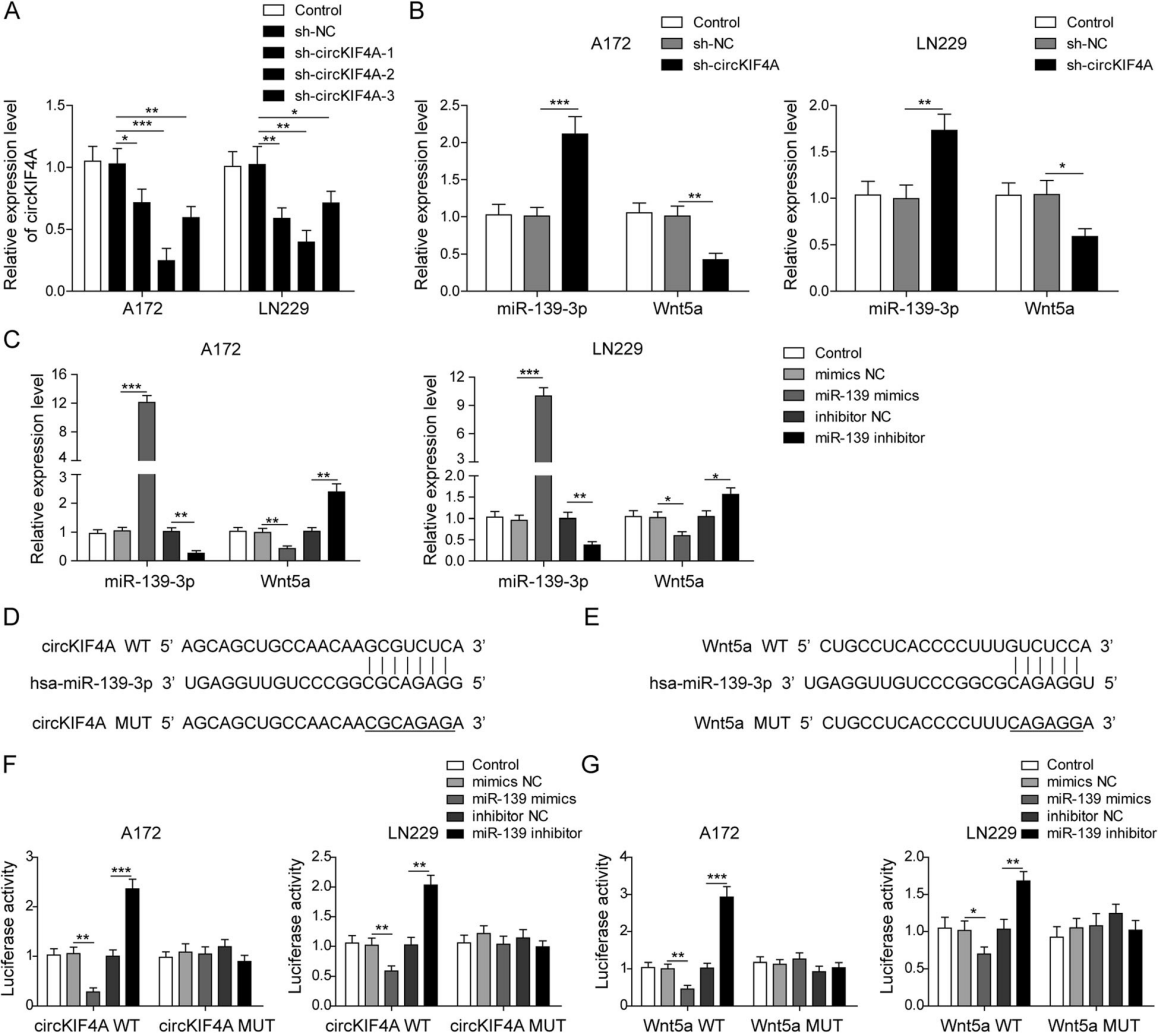
circKIF4A directly targeted miR-139-3p to promote the expression of Wnt5a in A172 and LN229 cells. **a** The expression level of circKIF4A was detected by qRT-PCR in A172 and LN229 cells transfected with sh-circKIF4A-1, sh-circKIF4A-2 and sh-circKIF4A-3. **b** miR-139-3p and Wnt5a were detected by qRT-PCR in A172 and LN229 cells transfected with sh-circKIF4A-2. **c** The expression level of miR-139-3p and Wnt5a were detected by qRT-PCR in A172 and LN229 cells transfected with miR-139-3p mimics or inhibitor. **d** Schematic representation of binding sites between circKIF4A and miR-139-3p predicted by StarBase software. **e** Schematic representation of binding sites between miR-139-3p and Wnt5a predicted by StarBase software. **f** Luciferase activity assay was performed after co-transfection with circKIF4A reporter plasmid and miR-139-3p into A172 and LN229 cells. **g** Luciferase activity assay was performed after co-transfection with Wnt5a reporter plasmid and miR-139-3p into A172 and LN229 cells. *p < 0.05, **p < 0.01, ***p < 0.001

To explore the regulatory relationship between circKIF4A, miR-139-3p and Wnt5a, the expressions of circKIF4A, miR-139-3p and Wnt5a were examined in A172 and LN229 cell lines which knocking down the circKIF4A. As shown in Fig. 2a, among A172 and LN229 cells transfected with sh-circKIF4A-1, sh-circKIF4A-2 and sh-circKIF4A-3 respectively, sh-circKIF4A-2 had the best knockdown efficiency. The downregulation of circKIF4A dramatically promoted the level of miR-139-3p and inhibited the level of Wnt5a (Fig. 2b). Next, the A172 and LN229 cell lines were transfected with miR-139-3p mimics or inhibitor, and the mRNA expression of Wnt5a were detected. Results showed that miR-139-3p mimics down-regulated the level of Wnt5a, while miR-139-3p inhibitor up-regulated the level of Wnt5a (Fig. 2c). We found the binding site of miR-139-3p in circKIF4A sequence by StarBase software, and Wnt5a may be the potential target of miR-139-3p (Fig. 2d, e). Subsequent luciferase reporter assays showed that luciferase intensity decreased/increased was observed after co-transfection of the wild type circKIF4A luciferase promoter and a miR-139-3p mimics/inhibitor, while the mutated luciferase reporter did not play this role (Fig. 2f). This indicated that circKIF4A directly interacts with miR-139-3p. Another luciferase reporter assay showed that the co-transfection of miR-139-3p mimics/inhibitor and Wnt5a-WT strongly decreased/increased the luciferase activity, but the co-transfection of miR-139-3p mimics/inhibitor and Wnt5a-MUT did not change it (Fig. 2g). This suggested that Wnt5a was a direct target gene of miR-139-3p. All the above results indicated that circKIF4A regulates Wnt5a expression by targeting miR-139-3p in glioma cells.

### circKIF4A knockdown inhibits the proliferation and promotes the apoptosis of glioma cells by regulating miR-139-3p

**Fig. 3:**
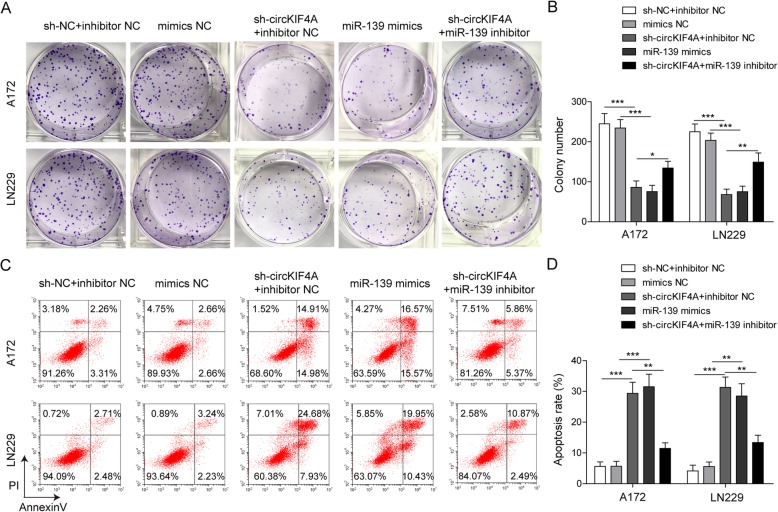
circKIF4A knockdown inhibited proliferation and promoted apoptosis via miR-139-3p in A172 and LN229 cells. After transfecting cells with sh-circKIF4A, miR-139-3p mimics or miR-139-3p inhibitors, **a** A colony formation assay to detect A172 and LN229 cells colony-forming ability. **b** The colony formation number was quantified by Image J. **c** The A172 and LN229 cells apoptotic rate was determined by flow cytometry. **d** Quantitative analysis of cell apoptotic rate. *p < 0.05, **p < 0.01, ***p < 0.001

Although the interaction between circKIF4A and miR-139-3p has been confirmed, the biological function of circKIF4A and miR-139-3p in glioma cells need to be well defined. The colony formation assay showed that circKIF4A knockdown or miR-139-3p mimics inhibited the colony formation in A172 and LN229 cells (Fig. 3a, b). At the same time, the flow cytometry assay showed that circKIF4A knockdown or miR-139-3p mimics dramatically promoted the apoptosis of glioma cells (Fig. 3c, d). To investigate the role of circKIF4A in regulating miR-139-3p on gliomas, a series of rescue experiments were performed. A172 and LN229 cells were co-transfected with sh-circKIF4A interference plasmid and miR-139-3p inhibitor, and their effects on colony formation and apoptosis were observed. Colony formation analysis showed that inhibition of cell proliferation induced by down-regulation of circKIF4A was reversed by miR-139-3p inhibitor (Fig. 3a, b). The flow cytometry assay revealed that sh-circKIF4A increased cell apoptosis could be rescued by miR-139-3p inhibitor (Fig. 3c, d). These results suggested that circKIF4A regulates cell proliferation and apoptosis through targeting miR-139-3p.

### circKIF4A knockdown inhibits the migration and invasion of glioma cells by targeting miR-139-3p

**Fig. 4:**
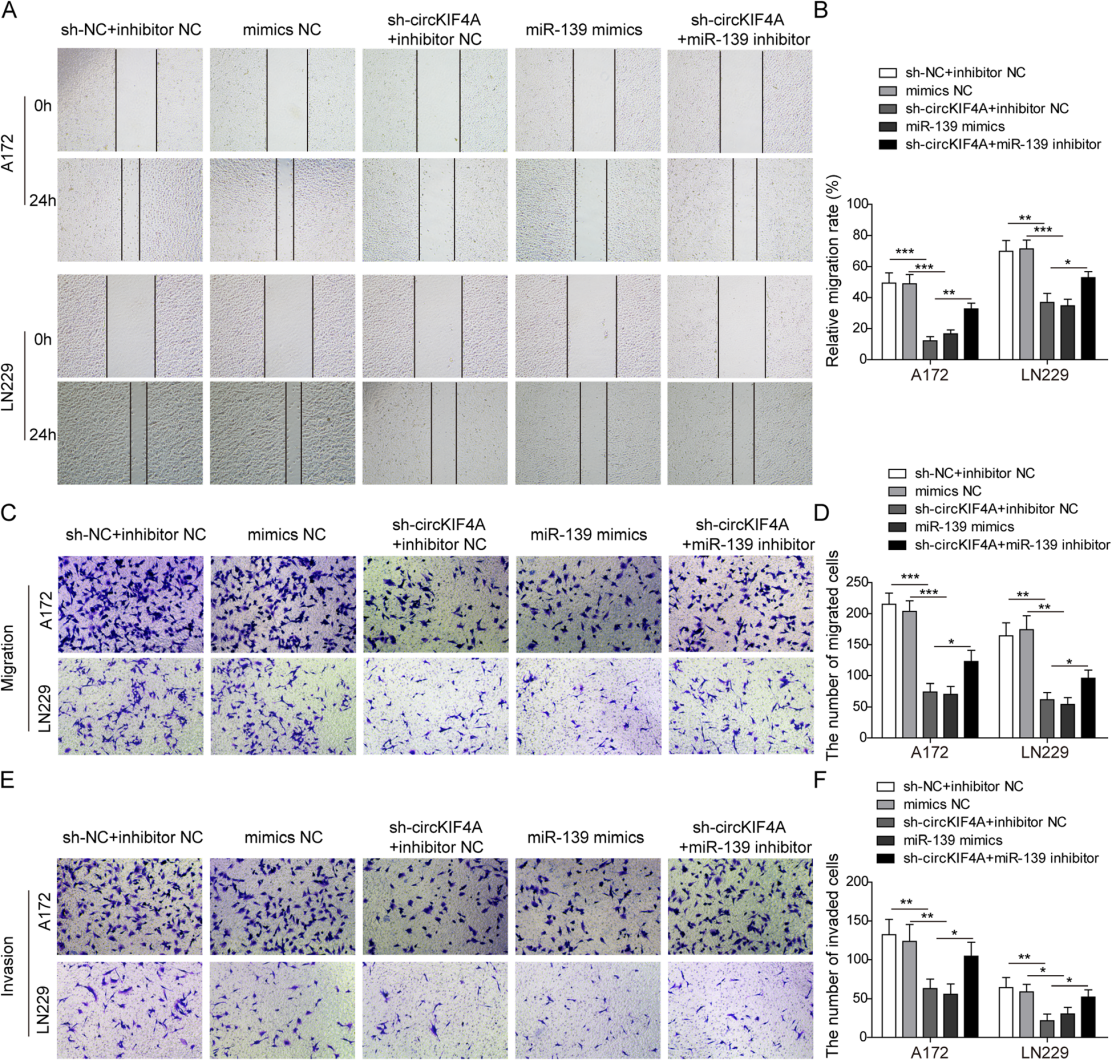
circKIF4A knockdown inhibited migration and invasion via miR-139-3p in in A172 and LN229 cells. After transfecting cells with sh-circKIF4A, miR-139-3p mimics or miR-139-3p inhibitors, **a** the migration of A172 and LN229 cells was determined by wound healing assay. **b** Quantitative analysis of cell migration rate. **c** The migration of A172 and LN229 cells was determined by transwell assay. **d** Quantitative analysis of the migration cell number. **e** The invasion of A172 and LN229 cells was determined by transwell assay. **f** Quantitative analysis of the invasion cell number. *p < 0.05, **p < 0.01, ***p < 0.001

To determine whether the effects of circKIF4A were mediated by miR-139-3p, we also detected the cell migration and invasion after circKIF4A and miR-139-3p alterations. Wound-healing assays showed that circKIF4A knockdown or miR-139-3p mimics inhibited the cell migration in A172 and LN229, as evidenced by the narrow wound gap at 0 h and 24 h compared with the control groups. At the same time, inhibition of cell migration induced by down-regulation of circKIF4A was reversed by miR-139-3p inhibitor (Fig. 4a, b). As expected, we observed the similar results in the transwell migration assays (Fig. 4c, d). Transwell invasion assays also showed that invasion numbers of A172 and LN229 cells were markedly decreased after sh-circKIF4A or miR-139-3p mimics transfected. Additionally, the alteration in cell invasion induced by circKIF4A knockdown was alleviated by miR-139-3p inhibitor (Fig. 4e, f). These results suggested that knockdown of circKIF4A inhibits cell migration and invasion of glioma cells through regulating miR-139-3p.

### circKIF4A knockdown represses Wnt/β-catenin signaling pathway by targeting miR-139-3p in glioma cells

**Fig. 5:**
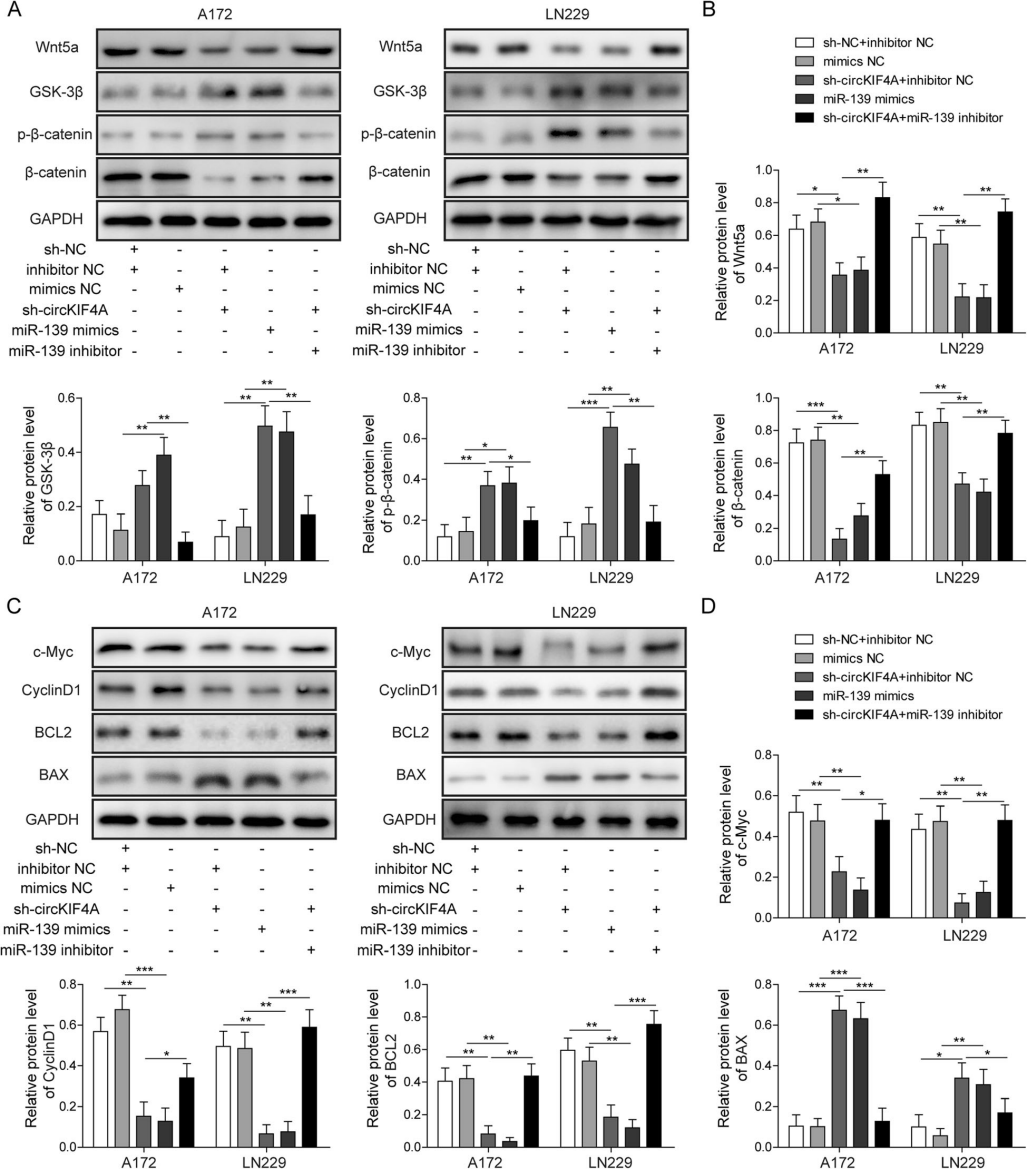
circKIF4A knockdown inhibited the Wnt/β-catenin signal pathway and regulated proliferation-related signal by targeting miR-139-3p. After transfecting A172 and LN229 cells with sh-circKIF4A, miR-139-3p mimics or miR-139-3p inhibitors, **a** the protein expression of Wnt5a, GSK-3β, β-catenin and p-β-catenin were detected by Western blotting. **b** The quantification of Wnt5a, GSK-3β, β-catenin and p-β-catenin relative protein level as a ratio to that of GAPDH (internal control). **c** The protein expression of c-Myc, cyclin D1, Bcl2 and Bax were detected by Western blotting. **d** The quantification of c-Myc, cyclin D1, Bcl2 and Bax relative protein level as a ratio to that of GAPDH (internal control). *p < 0.05, **p < 0.01, ***p < 0.001

To further investigate the molecular mechanisms of circKIF4A and miR-139-3p in glioma cells, the activities of Wnt/β-catenin signaling pathway were detected in A172 and LN229 cells. circKIF4A knockdown or miR-139-3p mimics inhibited the expression of Wnt5a and β-catenin, and promoted the expression of GSK-3β and p-β-catenin (Fig. 5a, b). The rescue experiments demonstrated that the expression changes of Wnt/β-catenin signaling pathway-related proteins induced by sh-circKIF4A were also significantly reversed by miR-139-3p inhibitors (Fig. 5a, b). Subsequently, we further examined the level of some proliferation-related and apoptosis-related proteins by Western blotting. The results revealed that circKIF4A knockdown inhibited the expression of c-Myc, cyclin D1 and Bcl2, and promoted the expression of Bax (Fig. 5c, d). miR-139-3p mimics showed consistent results with sh-circKIF4A in A172 and LN229 cells (Fig. 5c, d). After co-transfection of sh-circKIF4A with miR-139-3p inhibitor, proliferation and apoptosis-related protein expression changes induced by circKIF4A knockdown were partially abolished (Fig. 5c, d). These results suggested that knockdown of circKIF4A suppresses cell proliferation, apoptosis, migration and invasion of glioma cells through regulating miR-139-3p to inactivate Wnt/β-catenin signaling pathway.

### circKIF4A knockdown inhibits the spheres formation ability of GICs through miR-139-3p/Wnt5a signaling

**Fig. 6:**
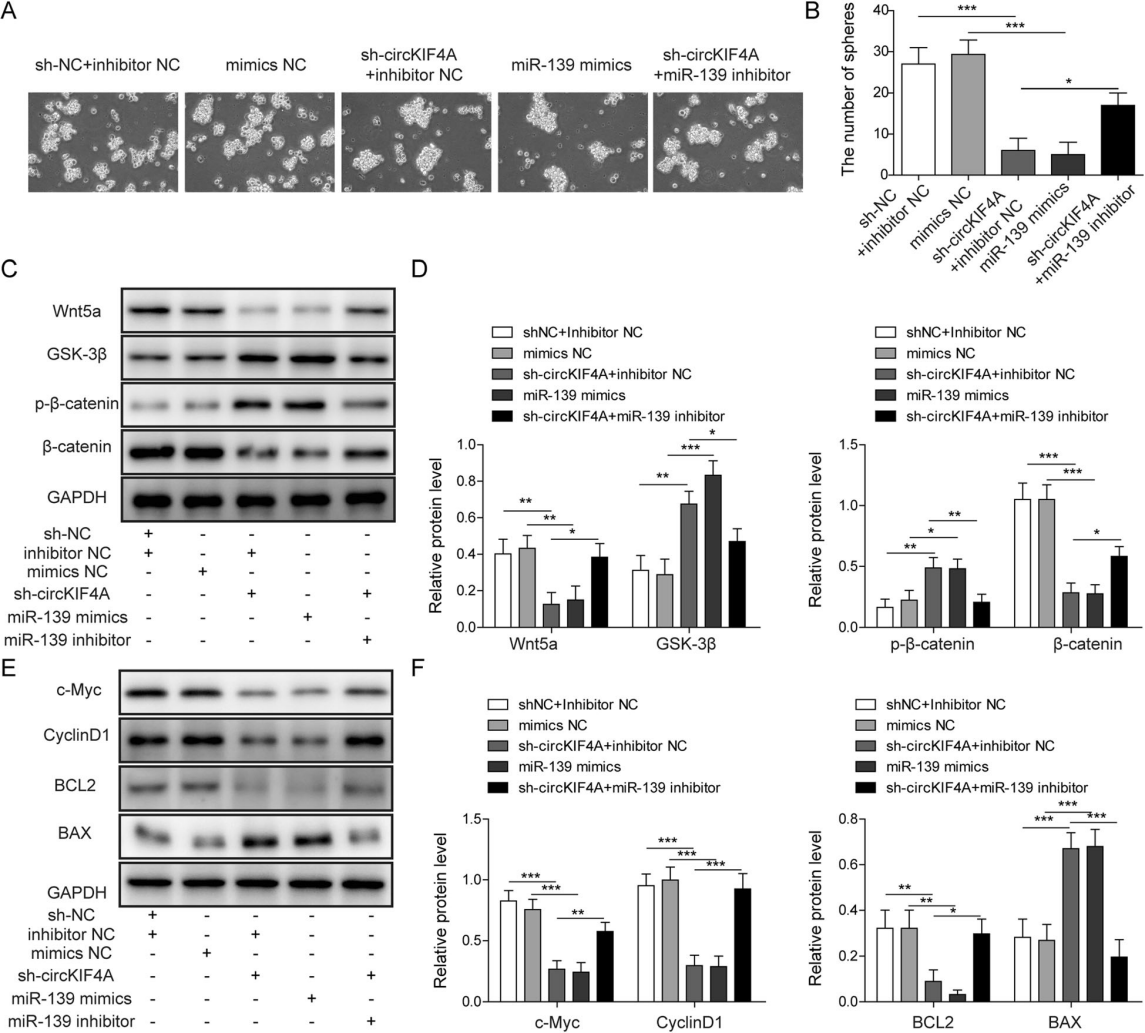
circKIF4A knockdown suppressed the spheroid formation of GICs through miR-139-3p/Wnt5a signaling. After transfecting GICs with sh-circKIF4A, miR-139-3p mimics or miR-139-3p inhibitors, **a** the sphere growth of GICs was determined by sphere formation assays. **b** Quantitative analysis of the GICs sphere number. **c** The protein expression of Wnt5a, GSK-3β, β-catenin and p-β-catenin were detected by Western blotting. **d** The quantification of Wnt5a, GSK-3β, β-catenin and p-β-catenin relative protein level as a ratio to that of GAPDH (internal control). **e** The protein expression of c-Myc, cyclin D1, Bcl2 and Bax were detected by Western blotting. **f** The quantification of c-Myc, cyclin D1, Bcl2 and Bax relative protein level as a ratio to that of GAPDH (internal control).*p < 0.05, **p < 0.01, ***p < 0.001

Studies have found that glioma is maintained by a GIC that is tumorigenic, therapeutically resistant, and recurrent (Hossain et al. 2015). Therefore, we wanted to determine the potential role of circKIF4A in the development and maintenance of the stem cell-like properties of GIC. We performed sphere formation experiments and found that the knockdown of circKIF4A expression in GICs resulted in a decrease in their ability to form spheres. At the same time, overexpression of miR-139-3p also impaired the ability of GICs to form spheres. Interestingly, inhibiting miR-139-3p could partially restore the reduced ability of GICs spheroid formation caused by circKIF4 knockdown (Fig. 6a, b). In addition, knockdown of circKIF4A or overexpression of miR-139-3p could promote the expression of GSK-3β, p-β-catenin and the pro-apoptotic protein Bax, and inhibit Wnt5a, β-catenin and the proliferation-related proteins CyclinD1, c-Myc, and Bcl-2 expression. While simultaneously inhibiting miR-139-3p could significantly abolish the expression changes of the aforementioned proteins caused by knockdown of circKIF4A (Fig. 6c-f). These results indicated that the down-regulation of circKIF4A inhibits the spheres formation of GICs and proliferation-related signals by regulating the miR-139-3p/Wnt5a signaling pathway.

### circKIF4A knockdown suppresses glioma cells tumorigenesis by regulating miR-139-3p/Wnt5a axis in vivo

**Fig. 7:**
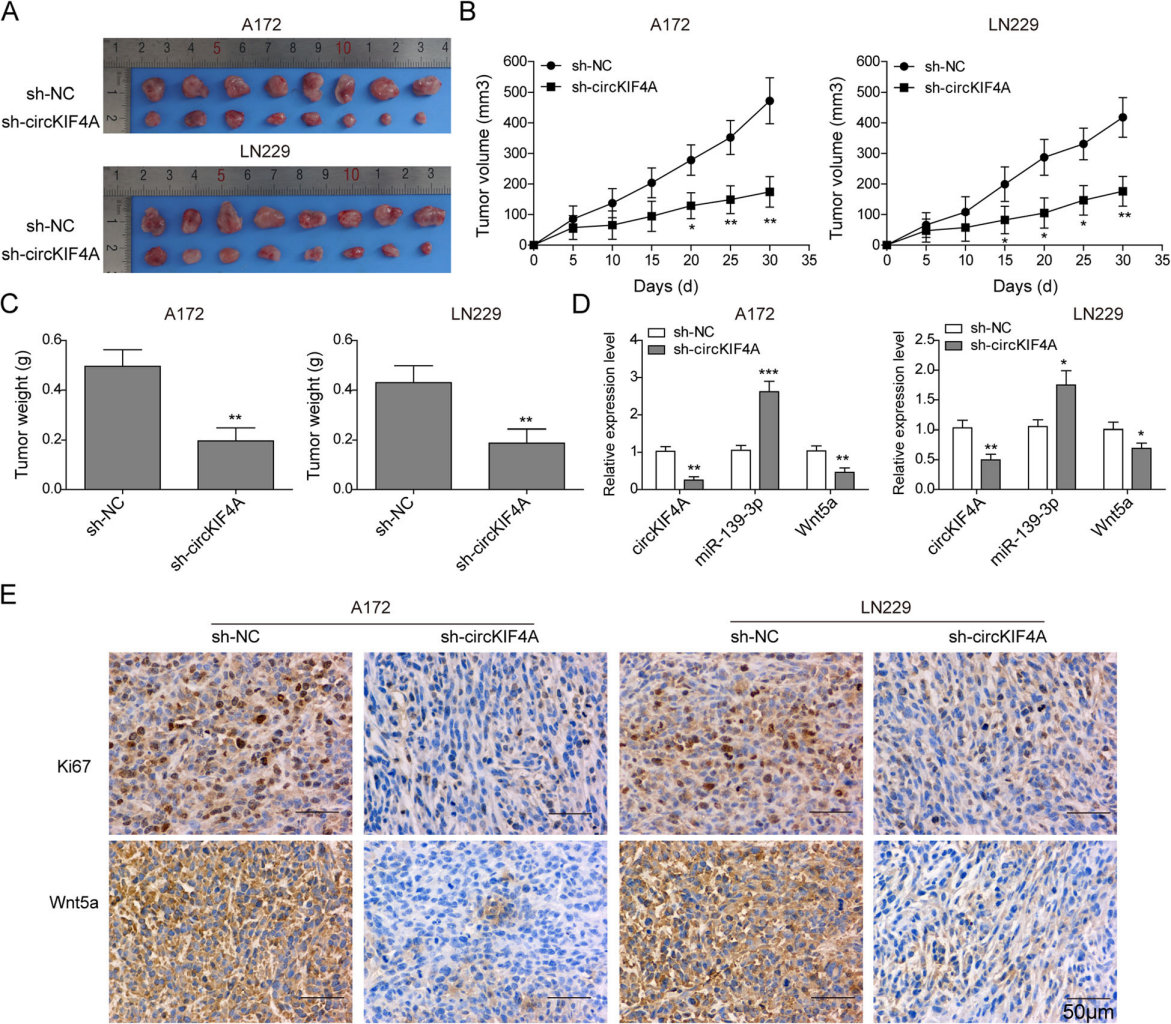
circKIF4A knockdown suppressed glioma cells tumorigenesis in nude mice. A172 and LN229 cells stably expressing sh-circKIF4A were injected into the nude mice. **a** Representative pictures of isolated xenografts were shown. **b** The tumor volume of each mouse was measured every 5 days. **c** 30 day later, the tumor weight of each group mouse was measured. **d** The expression level of circKIF4A, miR-139-3p and Wnt5a was measured by qRT-PCR. **e** Ki67 and Wnt5a protein level was detected by immunohistochemical staining. *p < 0.05, **p < 0.01, ***p < 0.001

To investigate the impact of circKIF4A on glioma tumorigenesis in vivo*,* A172 and LN229 cells of stably expressing sh-circKIF4A were injected into nude mice. Tumor volumes of each mouse were measured every 5 days and sacrificed 30 days later. As showed in Fig. 7a-c, circKIF4A knockdown decreased the tumor volume and weight compared with negative control. Moreover, the expression of miR-139-3p was up-regulated and Wnt5a was down-regulated in tumor tissues from sh-circKIF4A group, compared with that in sh-NC group (Fig. 7d). The immunohistochemical results showed that circKIF4A silencing significantly inhibited the expression of Ki-67 and Wnt5a compared with sh-NC group (Fig. 7e). These results revealed that circKIF4A regulates tumorigenesis of glioma in nude mice through Wnt/β-catenin signaling pathway.

## Discussion

Gliomas are the most common primary brain tumors and are currently treated with surgical resection, radiotherapy, and chemotherapy, but leading inevitably to severe disability and mortality outcomes (Gandia-Gonzalez et al. 2019). Glioma has been studied in numerous molecular studies aimed at developing new therapeutic strategies, while the overall survival rates of high-grade gliomas remain pessimistic (Liu et al. 2019b). Therefore, identifying the underlying mechanisms of glioma development and finding new therapeutic targets is an urgent issue. A growing body of evidence that circRNAs are involved in the development of glioma as an oncogene or tumor suppressor (Xie 2018; He et al. 2018; Barbagallo et al. 2018). It has been reported that circKIF4A expression is significantly increased in glioma tissues. However, the role and potential mechanism of circKIF4A in glioma progression remain unclear. In this study, we elucidated the role and regulatory mechanism of circKIF4A in glioma progression. Our results indicated that circKIF4A expression was dramatically increased while miR-139-3p was decreased in glioma tissues and cell lines. circKIF4A suppression reduced glioma cells colony formation ability, migration, invasion and increased apoptosis. circKIF4A inhibition could decrease Wnt5a expression by sponging miR-139, which repressed Wnt/β-cetanin signaling pathway. Furthermore, circKIF4A knockdown impaired the self-renewal of GICs and suppressed glioma cells tumorigenesis in nude mice. Therefore, we conclude that circKIF4A maybe a carcinogenic circRNA in glioma progression.

As a special endogenous non-coding RNA, circular RNA can be used as a competitive endogenous RNA (ceRNA) to participate in the regulation of gene expression, and it has become a new research hotspot after miRNA and long noncoding RNA (lncRNA) (Wilusz and Sharp 2013). Some studies reported that circRNA might act as sponges for miRNA, regulating the expression of miRNA target genes in glioma. Wang R et al. found that circNT5E could promote cell proliferation, migration, and invasion through acting as a sponge of miR-422a (Wang et al. 2018b). Li X et al. found that circ_0001946 acted as a competing endogenous RNA to modulating miR-671-5p and CDR1, and reduced proliferation, migration, invasion and increased apoptosis in GBM cells (Li and Diao 2019). To determine the expression status of circRNAs in GBM tissues, researchers conducted microarray assay of paired GBM tissues and displayed the top 20 up-regulated and down-regulated circRNAs, in which circKIF4A level was found to be significantly increased in glioma tissues (Wang et al. 2018a). At present, there are not many studies on circKIF4A in tumors. A recent study found that circKIF4A regulates triple-negative breast cancer progression by sponging miR-375 (Tang et al. 2019). Our study confirms that circKIF4A was up-regulated in gliomas, and knockdown of circKIF4A inhibits glioma cell proliferation, migration, invasion and xenograft growth in vivo. These results fully demonstrated that circKIF4A plays a key role in glioma tumorigenesis and can promote the development of glioma.

MiRNAs, the short non-protein coding RNAs, suppress target gene expression primarily by binding to 3′-untranslated region (3′-UTR) of mRNA in a Dicer-dependent manner (Guo et al. 2019). Dysregulation of certain miRNAs could contribute to the pathogenesis of various tumors. Previous studies demonstrated that miR-139-3p plays critical role in tumor progression. Zou ZC et al. showed that miR-139-3p inhibited tumor growth and metastasis in hepatocellular carcinoma by down-regulating ANXA2R expression (Zou et al. 2018). Huang P et al. suggested that miR-139-3p can inhibit cervical cancer cell migration invasion and proliferation by targeting NOB1 (Huang et al. 2016). Xia Z et al. showed that lncRNA-TP73-AS1 targets miR-139-3p to promote retinoblastoma cell proliferation (Xia et al. 2019). According to recent reports, the expression of miR-139-3p in human glioma samples was markedly lower than that in normal brain tissues, and miR-139-3p inhibited proliferation, migration and invasion of glioma cells (Tian et al. 2019; Shi et al. 2019). However, there is limited research on the molecular mechanism of miR-139-3p regulation of glioma development. In this study, we confirmed that the level of miR-139-3p was decreased in glioma cell lines and tissues. miR-139-3p mimics inhibit cell proliferation, migration, invasion, and induce apoptosis in A172 and LN229. The expression of miR-139-3p and circKIF4A was negatively correlated in glioma specimen. Furthermore, dual-luciferase reporter assay suggested that circKIF4A interacts directly with miR-139-3p in A172 and LN229 glioma cells. Importantly, a series of rescue experiments have shown that the changes of proliferative, apoptosis, migration and invasion induced by sh-circKIF4A were reversed by miR-139-3p inhibitors. Thus, circKIF4A maybe play an oncogenic role through targeting miR-139-3p in glioma.

miRNAs are known to regulate the progression of many diseases by downregulating the expression of target genes (Bartel 2009). Our study found a negative correlation between miR-139-3p and Wnt5a levels in glioma samples. Subsequently, it was proved that Wnt5a was the target gene of miR-139-3p in glioma cells. Wnt5a is an important member of the Wnt family, which plays an important role in cell differentiation, maturation and tumorigenesis. Wnt5a has dual functions of promoting or inhibiting cancer progression, and can play different roles depending on the type of cancer and the background of the receptor (McDonald and Silver 2009; Asem et al. 2016). For example, Wnt5a was found to be associated with decreased overall survival in patients with glioblastoma (Zeng et al. 2018), which was considered to have oncogenic potential. Consistent with previous reports, our study also found that Wnt5a level was significantly increased in glioma cell lines and tissues. Up-regulation of Wnt5a can induce Wnt/β-catenin activation (Peng et al. 2018; Bo et al. 2013). The mechanism by which Wnt signaling pathway regulating tumorigenesis involves many aspects such as signal transduction, cell cycle, cell proliferation migration and apoptosis (He et al. 2019). The abnormal activation of this pathway initiates the expression disorder of downstream target genes such as c-Myc and cyclin D1. c-Myc protein has the dual function of stimulating cell proliferation and regulating apoptosis (Ciribilli et al. 2015; Di Giacomo et al. 2017). Increased β-catenin in cytoplasm activates cyclin D1 which is key regulators of cell cycle promoting cell division, leading to uncontrolled cell proliferation and carcinogenesis (Mo et al. 2019). We observed that Wnt5a, β-catenin, c-Myc, cyclin D1 and anti-apoptotic Bcl-2 was decreased, whereas pro-apoptotic Bax were increased when circKIF4A was knockdown, it was important that miR-139-3p inhibitor could reduce the influence of sh-circKIF4A on the expression of the above proteins. Therefore, this study indicated that circKIF4A acts as a ceRNA to active Wnt5a/β-catenin signaling pathway mediated glioma progression by depressing miR-139-3p expression.

Finally, we found that silencing circKIF4A inhibited spheroid formation of GICs through miR-139-3p/Wnt5a signaling. GICs are characterized by their self-renewal ability and tumorigenicity and have been identified as a highly tumorigenic subpopulation of glioblastoma multiforme, which is considered to be a recurrence of glioblastoma and causes of chemical/radiation resistance (Xia et al. 2013). Because GICs plays a central role in the tumorigenicity of glioblastoma, a large amount of research in recent years has focused on targeting GICs as a new glioblastoma therapeutic target. Several research reports have revealed that Wnt/β -catenin signaling activation is essential for GICs self-renewal. For example, Sachin S Rathod et al. found that miR-34a inhibited GICs cell proliferation and tumor growth by targeting the Wnt signaling pathway (Rathod et al. 2014). Zheng et al. found that PLAGL2 regulated GICs proliferation and glioma development through Wnt signaling (Zheng et al. 2010). Our results are similar to these studies, suggesting that circKIF4A may play a role in the maintenance of GICs through the Wnt/β-catenin signaling pathway.

## Conclusions

In summary, we found a significant increase in the expression of circKIF4A in gliomas. The circKIF4A gene knockdown decreased the colony formation ability, migration and invasion of glioma cells, and reduced tumor growth in vivo. In terms of mechanism, we demonstrated that low expression of circKIF4A suppressed GICs self-renewal and glioma progression via modulating miR-139-3p/Wnt5a/β-catenin axis. These findings may provide a new therapeutic strategy for glioma.

## Data Availability

All data generated or analysed during this study are included in this published article [and its supplementary information files].

## Abbreviations

GICs: Glioma-initiating cells
WHO: World Health Organization
LGG: Low-grade gliomas
HGG: High-grade glioma
GBM: Glioblastoma multiforme
FZD: Frizzleds
Dvl: Disheveled
TCF/LEF: T cell factor/lymphoid enhancer-binding factor
NHAs: Normal human astrocytes
